# Increased sleep duration precedes the improvement of other symptom domains during the treatment of acute mania: a retrospective chart review

**DOI:** 10.1186/s12888-016-0808-7

**Published:** 2016-04-12

**Authors:** Igor I. Galynker, Zimri S. Yaseen, Siva S. Koppolu, Barney Vaughan, Magdalena Szklarska-Imiolek, Lisa J. Cohen, Thomas M. Salvanti, Hae-Joon Kim

**Affiliations:** Mount Sinai Beth Israel Medical Center, Psychiatry and Behavioral Sciences, 317 E 17th St., 9 Fierman Hall, New York, NY 10003 USA

**Keywords:** Bipolar disorder, Sleep, Psychosis, Mania

## Abstract

**Background:**

Understanding trajectories of symptom changes may help gauge treatment response and better identify therapeutic targets in treatment of acute mania. We examined how symptoms of sleep disturbance, mania, and psychosis resolved in a naturalistic treatment setting, hypothesizing that improvement in sleep would precede improvement in manic and psychotic symptoms.

**Methods:**

Charts of 100 patients with admitting diagnoses of bipolar mixed or manic episode were retrospectively reviewed. Medications and demographic variables were recorded, and the Clinician-Administered Rating Scale for Mania (CARS-M) mania and psychosis ratings and sleep hours were determined for 8 observation points. Times to minimum symptom level in each domain were compared via Wilcoxon signed-rank tests. Symptom correlations and trajectories and medication effects were explored using repeated measures ANOVA and regression models.

**Results:**

Manic and psychotic symptom resolution was linear over the time of hospitalization. In contrast, sleep showed a slow initial response, followed by rapid increase to peak, preceding peak improvement in mania and psychosis (*p* < 0.001). Rate of sleep restoration was a predictor of rate but not of magnitude of treatment response for symptoms mania and psychosis. Patterns of medication use did not affect symptom trajectories.

**Conclusions:**

In acute mania, improvement in sleep with treatment is dissociable from resolution in symptoms of mania and psychosis, but there appears to be no therapeutic advantage to patient oversedation. Sleep improves first and may be both a predictor of the rate of treatment response and a useful therapeutic target.

## Background

Bipolar disorder is a disorder of mood, which is distinguished from other mood disorders by manic episodes – sustained periods of decreased sleep and increased energy, as well as elevated or irritable mood and, in some instances, psychosis. The extent to which the symptom domains of sleep disturbance [1, 2], mood, and psychosis are dissociable processes is unclear. Knowledge of the relationships between and trajectories of these symptom domains may help better identify therapeutic targets as a manic episode progresses from its onset to its resolution.

Studies indicate that sleep disturbance plays a prominent role in the prodromal phase of bipolar disorder [3, 4]. Animal models indicate that sleep deprivation may trigger a manic episode [5], while in humans, chronotherapy with sleep deprivation is effective in elevating mood in depression (via the reset of circadian rhythms disrupted by CLOCK gene dysfunction) as well as in depression with psychotic features [6, 7]. Several studies have suggested that sleep disturbance may be a possible mechanism for future illness relapse [8, 9], and a recent review cited sleep deprivation as a main trigger of an episode switch [10]. A recent study found that poor sleep quality continues during euthymia, and that sleep disturbance may be a prodromal sign or trigger of a change in mood episode [11]. Finally, studies examining environmental disturbances to circadian/social rhythms’ contributions to mania onset suggest they do so via sleep disturbance [12].

In contrast to the relative abundance of studies on the associations between sleep disturbances and onset of mania [13–16], we are aware of just one study, a small retrospective chart review, attempting to assess the temporal relationship between sleep restoration and resolution of mania [17]. Finally, one double-blind comparison of the effect of the antipsychotics haloperidol and olanzapine on sleep in mania [18] noted differential effects of these drugs on multiple sleep measures as well as different trajectories of improvement in sleep and manic symptoms or psychosis, suggesting a possible influence of sleep-symptom treatment on the course of mania.

A number of studies examined the relationship between psychosis and sleep without regard to symptoms of mania. Individuals at-risk for psychosis compared to healthy controls had poorer sleep quality, more fragmented sleep rhythms, and circadian rhythms that were not synchronized with dark–light cycles, suggesting a link between sleep and the onset of psychotic disorders [19, 20]. Early studies found that sleep variables such as decreased REM sleep and REM activity differentially affected psychotic and nonpsychotic depressive patients, and increased sleep hours and REM activity accompanied improvement in psychotic symptoms [7, 21]. Finally, a more recent study on the relationship between psychopathology and sleep in schizophrenia found that a change in patterns of sleep preceded any changes in symptom severity [22].

Understanding trajectories of improvement in sleep and mania during treatment of acute manic episodes may inform treatment decisions. Specifically, since sleep disturbance is prodromal to mania onset, partial or even complete resolution in manic symptoms without sleep restoration may indicate continuing acute manic episode warranting further aggressive treatment, despite symptomatic improvement. Similarly, if sleep restoration precedes and is correlated with improvement in symptoms of mania, sleep duration may present a useful index of treatment progress and an easily quantifiable therapeutic target. Similar considerations would apply to the relationship of mood and sleep domains with psychosis.

In this context we sought to examine how the symptom domains of sleep, mania, and psychosis resolve during naturalistic inpatient treatment and test the following three hypotheses regarding the trajectories of symptom resolution: i) as in manic episode onset, sleep disturbance during episode resolution would be dissociable from symptoms of mania and psychosis, ii) that sleep would improve faster than symptoms of the other two domains and the sleep improvement rate would predict the rates of improvement in mania and psychosis, rather than the reverse – that symptom changes might precede improvement in sleep given that hyperarousal or rumination due to psychosis and mania might affect sleep, and iii) that the magnitude of sleep disturbance at admission and magnitude of sleep improvements in the beginning of the hospitalization would predict the magnitude of manic and psychotic symptom severity at the end of hospitalization.

## Methods

This retrospective chart review study was approved by the institutional review board of the Beth Israel Medical Center of New York (BIMC). Charts were retrieved from computer storage using Access Anywhere software. All inpatient psychiatric admissions to BIMC from January 1^st^, 2003, to January 1^st^, 2010 were screened for a billing diagnosis of bipolar disorder, current episode manic or mixed state. The authors identified 100 completed clinical charts that fit the inclusion criteria. A chart was considered completed when it included a patient’s demographic information, psychiatric history, medication administration records, symptomatic assessments by nurses, resident psychiatrists and psychology interns, and supervising attending psychiatrists, records of any measures of isolation or restraint due to agitation, and duration of sleep. Sleep duration was computed from nurses’ notes based on their morning report of total hours of sleep for each patient, verified by chart records of their hourly checks throughout the night.

Sleep and symptom severity data were extracted from patient charts for each of eight time points: once upon the patient’s admission, each Monday, Wednesday and Friday (the days on which MD documentation in charts was mandatory) for the first two weeks following admission, and once at discharge, following the schedule on which psychiatrists write inpatient notes at BIMC.

Demographic information including patient age, sex, race, substance use/abuse status, and length of stay were extracted from patient charts (See Table 1). The medications administered to each patient were also extracted from patients’ PRISM software medication administration records throughout the entirety of hospitalization. Antipsychotics were classified as first or second generation and were converted to chlorpromazine milligram equivalent doses, while benzodiazepine doses were converted to lorazepam milligram equivalents (See Table 2). Diphenhydramine was the only antihistamine administered and milligram doses administered were recorded.

**Table 1:** Sociodemographic characteristics and medications of 100 bipolar inpatients admitted with manic or mixed episode

Variables	n	%	Mean ± SD
Age (years)			40.5 ± 13.5
Sex			
Male	52	52.0	
Female	48	48.0	
Race/Ethnicity			
Caucasian	26	26.0	
African American	15	15.0	
Hispanic	7	7.0	
Asian	4	4.0	
Middle Eastern	3	3.0	
Other	16	16.0	
Not recorded	29	29.0	
Length of hospital stay (days)^a^			31.21 ± 24.8
Number of previous psychiatric hospitalizations			1.4 ± 1.3
At least one substance use disorder^b^	51	51.0	
Alcohol use disorder	45	88.2	
Marijuana use disorder	31	60.8	
Cocaine use disorder	15	29.4	
Opiate use disorder	6	11.8	
Methadone maintenance	5	9.8	
Other substance use disorder	4	7.8	
Number of patients with complete medication records	89	89.0	
Number of medications per patient^c^			4.11 ± 1.77
First generation antipsychotics^b^	49	55.1	
Haloperidol	42	85.7	
Chlorpromazine	5	10.2	
Perphenazine	3	6.1	
Fluphenazine	3	6.1	
Loxapine	1	2.0	
Second generation antipsychotics^b^	76	85.4	
Olanzapine	28	36.8	
Risperidone	22	28.9	
Quetiapine	22	28.9	
Aripiprazole	10	13.2	
Ziprasidone	10	13.2	
Clozapine	2	2.6	
Paliperidone	1	1.3	
Mood stabilizers^b^	70	78.7	
Valproate	39	55.7	
Lithium	28	40.0	
Oxcarbazepine	5	7.1	
Lamotrigine	4	5.7	
Topiramate	2	2.9	
Gabapentin	1	1.4	
Carbamazepine	1	1.4	
Benzodiazepines^b^	69	77.5	
Lorazepam	58	84.1	
Clonazepam	33	47.8	
Diazepam	4	5.8	
Zolpidem	3	4.3	
Antihistamines (Diphenhydramine)	39	43.8	
Number of patients on any antipsychotic	88	98.9	
Number of patients on mood stabilizer and antipsychotic	68	76.4	

^a^5 % trimmed mean

^b^Percentages within category

^c^Mean calculated for patients with complete medication records (*n* = 89)

**Table 2:** Equivalent doses for antipsychotics and benzodiazepines

Drug	Milligrams of Chlorpromazine Equivalent to 1 of Drug
FGAs	
Haloperidol	100
Fluphenazine	100
Trilafon	25
Thioridazine	2
Loxapine	20
SGAs	
Risperidone	200
Olanzapine	40
Aripiprazole	40
Ziprasidone	10
Quetiapine	1.6
Clozapine	2.5
Drug	Milligrams of Lorazepam Equivalent to 1 of Drug
BZDs	
Diazepam	0.1
Clonazepam	2
Alprazolam	2

Raters extracted symptomatic severity assessments from doctors’, nurses’, social workers’, and occupational therapists’ chart entries using the Clinician Administered Rating Scale for Mania (CARS-M) [23]. The CARS-M is a 15-item observer-rated scale with two principle components, mania and psychosis, which have demonstrated strong inter-rater, test-retest, and internal reliability, and strong convergence with other rating scales for mania. The mania domain is measured by the first 10 items, which assess features like euphoria, irritability, hypermotor activity, pressured speech, flight of ideas, distractibility, grandiosity, excessive energy, poor judgment, and decreased need for sleep. Psychosis is measured by the final 5 items, which include disordered thinking, delusions, hallucinations, orientation, and insight. Each item is scored on a 5-point Likert scale, ranging from 0 to 5 (absent, slight, mild, moderate, severe, extremely severe), with descriptive clinical anchors for each level of severity > 0 [23].

The CARS-M symptom-related information on the charts was rated by three psychiatrists in their second through fourth year of residency training, under the direct supervision of IIG. Each item on the CARS-M at each time-point was rated by 3 raters, and, when the three independent ratings did not agree, least difference criteria were used to achieve a consensus score for each item as follows: If two clinicians gave the same score on any given data point, and one clinician gave a different score, the score given by the two agreeing clinicians was taken. If all three clinicians disagreed in a contiguous manner (e.g. 3, 4, and 5), the median score (4 in this case) was taken. If they disagreed in a noncontiguous manner (e.g. 2, 4, and 8), the mean of the two closest scores [3] was taken.

### Statistical analysis

To assess and compare trajectories of sleep, CARS-M mania, and CARS-M psychosis scores, repeated measures ANOVA was performed assessing each measure across observation days one through seven (See Table 3). In a secondary analysis, cumulative doses from observation days one through seven of benzodiazepine lorazepam equivalents, first and second generation antipsychotic chlorpromazine equivalents, and milligrams of diphenhydramine were added to the model as covariates to account for possible differential effects on sleep, mania, and psychosis. In addition, we considered sex and substance use as potential confounds.

**Table 3:** Results of repeated measures ANOVA

a. Within subjects univariate tests					
Measure	Type III Sum of Squares	df	Mean Square	F	Sig.
Sleep	84.88	6	14.15	5.55	<.001
Mania	4042.82	6	673.80	54.14	<.001
Psychosis	1117.91	6	186.32	44.68	<.001
b. Within subjects contrasts					
Measure	Type III Sum of Squares	df	Mean Square	F	Sig.
Sleep Linear	70.58	1	70.578	17.83	<.001
Sleep Quadratic	4.43	1	4.429	1.55	.22
Sleep Cubic	8.81	1	8.813	4.04	.05
Mania Linear	3960.19	1	3960.187	134.66	<.001
Mania Quadratic	58.65	1	58.647	4.61	.03
Mania Cubic	19.73	1	19.734	2.01	.16
Psychosis Linear	1117.49	1	1117.487	108.57	<.001
Psychosis Quadratic	.06	1	.056	.01	.92
Psychosis Cubic	.03	1	.028	.01	.92
c. Multivariate tests of between subjects effects					
Measure	Pillai’s Trace	df	Mean Square	F	Sig.
BZD cumulative dose	.14	78	3.00	4.36	.01
DPH cumulative dose	.06	78	3.00	1.61	.19
FGA cumulative dose	.05	78	3.00	1.33	.27
SGA cumulative dose	.05	78	3.00	1.21	.31
d. Multivariate tests of within subjects effects					
Measure	Pillai’s Trace	df	Mean Square	F	Sig.
Time	.56	63	18.00	4.40	<.001
BZD dose * Time	.32	63	18.00	1.62	.08
DPH dose * Time	.26	63	18.00	1.24	.26
FGA dose * Time	.19	63	18.00	.82	.67
SGA dose * Time	.21	63	18.00	.94	.54

Because observation day numbers are not a linear metric of time, we used paired non-parametric Wilcoxon signed-rank tests to compare time to maximum improvement (when the symptoms would reach greatest sleep hours, and lowest CARS-M subscale scores) over all observation days excluding observation day one. At maxima, increases in sleep and decreases in mania and psychosis measures could either peak or plateau: we use the term “peak” throughout for simplicity. Observation day one was excluded to eliminate the possibility of a patient arriving highly sedated from the ER or from another hospital as a transfer and thus having spuriously high sleep or low mania or psychosis scores. All statistical analyses were performed with SPSS 16.0.

To test prediction of symptom response by early sleep duration we examined univariate correlations between mean sleep on observation days one and two and mean symptom severity on observation days six and seven. To test prediction of symptom response by degree of sleep improvements early in hospitalization we examined univariate correlations between change in mean sleep (between mean sleep on observation days one and two and mean sleep on observation days four and five) and mean symptom severity on observation days six and seven. Two observation-day means were used to reduce noise in the data.

## Results

### Sample demographics

Patient mean age was 40.5 [13.5] (mean [SD]) years. Length of stay (LOS) in the hospital ranged from 10 to 189 days, with a 5 % trimmed mean LOS of 34.4 [24.8] days.

The number of previous psychiatric hospitalizations reported ranged from 0 to 4, with a mean of 1.4 [1.3] prior hospitalizations. In the sample of 100, 45 had no substance use disorder (SUD), 51 had at least one SUD, and 4 had missing SUD data. Of those with at least 1 SUD, 45/51 had an alcohol use disorder, 31/51 had a marijuana use disorder, 15/51 had a cocaine use disorder, 6/51 had an opiate use disorder, 5/51 were in a methadone maintenance treatment program, and 4/51 had other substance use disorders. Among 100 patients, 52 were male, and 26 were Caucasian, 15 African American, 7 Hispanic, 4 Asian, 3 Middle Eastern, 16 other race/ethnicity, and 29 did not have race/ethnicity recorded.

### Sample treatment characteristics

Complete medication administration data were available for 89/100 charts. The division of each medical class administered to 89 of the 100 patients during hospitalization was recorded as follows: 49 patients received first generation antipsychotics (FGAs), 76 received second generation antipsychotics (SGAs), 70 received mood stabilizers (MSs), and 69 received benzodiazepines (BZD).

In the group of patients who received SGAs, the most commonly used SGAs were Olanzapine, Risperidone, and Quetiapine, followed by Aripiprazole and Ziprasidone. Clozapine and Paliperidone were used least frequently. Among FGAs, the most commonly used was Haloperidol, followed by Chlorpromazine, Perphenazine, Fluphenazine, and Loxapine. Among mood stabilizers, the most commonly used were valproate and lithium. At baseline, CARS-M mania and psychosis scores were correlated with each other (*r* = 0.248, *p* = 0.013) but not with sleep (mania; *r* = −0.144, *p* = 0.159; psychosis: *r* = 0.011, *p* = 0.913).

### Sleep, mania, and psychotic symptom trajectories

**Fig. 1:**
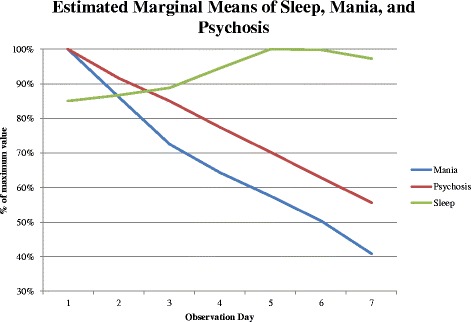
Trajectory of mania, psychosis, and sleep across 7 observations (admission through 2 weeks). Percentage of maximum sleep (hours) and percentage of maximum CARS-M mania and psychosis scores versus observation number

As expected, repeated measures ANOVA revealed significant improvements over time in sleep hours, mania, and psychosis scores (*p* < 0.001) for all measures (See Table 3a). Examination of the trajectories of sleep, mania, and psychosis indicated significant linear components for all measures with *p* < 0.001 (See Table 3b, Fig. 1).

**Fig. 2:**
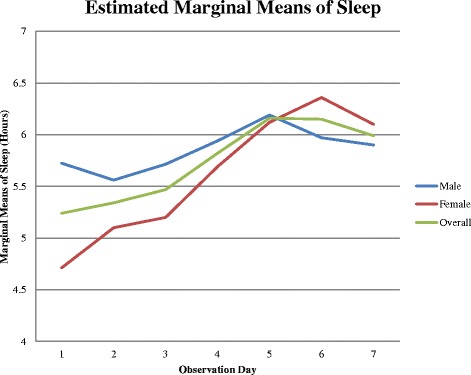
Trajectory of sleep across 7 observations (admission through 2 weeks). Estimated marginal means of sleep (hours) versus observation number

In addition, we found a significant cubic term for change in sleep (*F* = 4.04, *p* = 0.05), accounting for the generally sigmoidal trajectory (See Fig. 2) of initial slow improvement followed by rapid increase in sleep hours which then leveled off. For mania, we found a significant quadratic term (*F* = 4.61, *p* = 0.03), accounting for the lower rate of improvement in observations four through seven versus observations one through three. Maximal improvement in mean sleep was reached in observation day five, whereas maximal improvements in mean mania and psychosis scores were observed in observation day seven.

When the model was covaried for cumulative doses of sedating medications (benzodiazepines, first and second generation antipsychotics, and antihistamines) significant between subject differences were found only for cumulative benzodiazepine dose (*F* = 4.36, *p* = 0.01), however there were no significant interactions between cumulative doses and time in the over-all multivariate within-subjects model. Thus, in multivariate analysis, there were no significant changes in symptom trajectory associated with medication use (See Table 3c-da-b). Further, when the model was covaried for age, no significant effects were found.

In univariate analyses however, there were significant within subjects effects of cumulative benzodiazepine dose on mania trajectories (cumulative dose*observation day *F* = 2.119, *p* = 0.05). In other words, over the course of their hospitalizations, patients receiving higher cumulative benzodiazepine doses had more pronounced nonlinear distortions to their manic symptom trajectories, with tendencies toward higher initial levels of mania which improved more rapidly at first before plateauing.

In relation to sleep and psychosis, no medication showed significant between subjects effects. In relation to mania, only cumulative benzodiazepine doses showed significant between subjects effects (*F* = 12.899, *p* = 0.001), while cumulative antihistamine doses were marginally significant (*F* = 3.197, *p* = 0.078), both being higher in patients with higher mania scores. Further, when the presence or absence of any substance use or of any stimulant use were included in the model as between subjects factors, no significant effects were found either between or within subjects on any symptom measure.

### Sex effects on symptom trajectories

In a secondary analysis we found that sex had a marginally significant overall impact on sleep trajectory (*F* = 2.076, *p* = 0.054), with a significant linear effect in the within-subjects contrasts analysis (*F* = 7.579, *p* = 0.007), but not on mania or psychosis trajectories. In other words while there were no sex differences in mania and psychosis trajectories, on admission, sleep disturbance in men was less severe but improved more slowly than sleep disturbance in women (See Fig. 2).

### Relative times to peak improvement for sleep, mania, and psychosis

As hypothesized, paired Wilcoxon signed-rank tests revealed that the observation day during which patients achieved the maximum hours of sleep (mean observation day 4.69) significantly preceded the observation day during which they received the minima scores on the illness severity scales (mania trough mean observation day 6.98, *p* < 0.0005; psychosis trough mean observation day 7.3, *p* < 0.0005; CARS-M total score trough mean observation day 7.74, *p* < 0.0005). Shorter times to peak improvement in mania versus psychosis did not reach significance (paired Wilcoxon signed-rank test two-tailed *p* = 0.123).

There were no significant differences between men and women in time to peak improvement in any symptom domain, though time to peak improvement in mania scores was marginally lower for women than men (Mann–Whitney U two-tailed *p* = 0.1).

### Relation between sleep and manic and psychotic symptom responses

As expected, level of mania on observation day one was significantly correlated with level of mania averaged across observation days six and seven (*r* = 0.404, *p* < 0.0005) and severity of psychosis on observation day one was significantly correlated with severity of psychosis averaged across observation days six and seven (*r* = 0.407, *p* < 0.0005).

As hypothesized, time to peak sleep correlated significantly with time to lowest mania rating (Spearman’s rank *r* = 0.302, two-tailed *p* = 0.002) and with time to lowest psychosis rating (Spearman’s rank *r* = 0.265, two-tailed *p* = 0.008). In other words, rate of sleep restoration was a moderate predictor of rate of treatment response for symptoms of mania and psychosis.

There were no significant correlations between time to peak sleep and manic (rho = 0.13, *p* > 0.1) or psychotic symptom severity (rho = 0.16, *p* > 0.1) averaged across observation days six and seven (i.e., after approximately two weeks of hospitalization). Likewise, early sleep gains were uncorrelated with manic (rho = 0.11, *p* > 0.1) or psychotic (rho = −0.07, *p* > 0.1) symptom severity averaged across observation days six and seven. In other words early restoration of sleep was not a predictor of the magnitude of treatment response for symptoms of mania and psychosis.

## Discussion

This study examined the trajectories of and relationships between sleep, manic symptoms, and psychotic symptoms in bipolar patients hospitalized for a manic episode with the hope of identifying preferred therapeutic targets at different times in the course of treatment of mania. To our knowledge this is the first study to do so.

Our first hypothesis, that sleep disturbance would be dissociable from symptoms of mania and psychosis, was supported. Sleep resolution followed a sigmoid curve with initial slow improvement followed by a rapid change while mania and psychosis improvements were linear.

The results further indicated that sleep disturbance would resolve more rapidly than manic and psychotic symptoms, and that improvement in sleep would predict improvement in other domains, such that time to peak sleep would correlate with time to peak improvement in mania and psychosis. Thus our second hypothesis was also supported – maximal sleep improvements robustly preceded those in other domains and rate of sleep improvement was in this way a predictor of rate of improvement in manic and psychotic symptoms. The alternative to our second hypothesis, that symptom changes might precede improvement in sleep due to sleep disturbances caused by psychosis and mania was also partially supported, as some decline in manic and psychotic symptoms was observable prior to significant improvement in sleep duration. In short, our findings suggest that in treatment of mania, sleep should be assessed separately from both mania and psychosis and that sleep may be a useful prognostic guide in the treatment of manic episodes.

Our third hypothesis, that hours of sleep near time of admission and sleep improvement rates would predict the magnitude of improvement in manic and psychotic symptoms achieved over the first 2 weeks of hospitalization, was not supported. This finding suggests that more sleep does not mean less mania and psychosis. In other words, over-sedating patients does not result in fewer symptoms of mania and psychosis.

Our findings suggest that in treatment of mania, sleep needs to be monitored separately from mania and psychosis. Not unexpectedly, early severity in each domain was found to be the strongest predictor of the subsequent course in that domain. Still, although improvement in sleep and mania/psychosis are relatively independent processes, our data suggests that symptoms of mania and psychosis typically improve only after improvement in sleep, suggesting that early in treatment of acute mania, sleep may be both a good indicator of treatment progress and a good or even preferred treatment target.

Our finding that the sleep improvement curve is sigmoid, with initial relative persistence of sleep disturbance for about a week followed by rapid improvement and normalization by the middle of the second week, has important clinical implications. Due to the slow initial rate of sleep restoration, particularly in males, in many cases partial or even complete resolution in manic symptoms in the first week of treatment may precede sleep restoration, creating a misleading impression that the index manic episode has been successfully treated allowing hospital discharge.

Our results suggesting a sex difference in sleep disturbance and rates of sleep restoration are supported by a previous study on gender-specific sleep in bipolar disorder, which found that bipolar women reported poorer sleep quality, and poor sleep quality at episode onset predicted more severe and variable manic episodes [24]. On the other hand, our finding that sleep in men improved more slowly is new, and, if replicated suggests that sleep as a treatment target can be addressed and managed differently by gender.

However, regardless of gender, only normalization of both domains will reliably signify the end of the acute manic episode and warrant a switch to maintenance treatment of bipolar disorder. Thus, discharging patients having continuing sleep disturbance even with marked improvement in mania and psychosis means incomplete resolution of the acute manic episode which may result in a need for hospital readmission for further aggressive treatment.

Finally, our results finding a lack of correlation between the magnitude of sleep disturbance (or improvement) and the severity of symptoms of mania and psychosis indicate that excessive sleep may not be related to complete resolution of mania and psychosis. In other words, it appears that excessive sleep in treatment of mania may not necessarily be therapeutic.

These results should be considered in the light of several limitations, including the retrospective chart review design, and the use of clinical rather than standardized diagnoses. Further, CARS ratings were based on clinical documentation rather than direct patient observation. However, previous studies have demonstrated the feasibility of making clinical ratings based on psychiatric medical records [25, 26], and the combination of multiple observer data and multiple rater scoring with concrete descriptive anchor points for symptom severity ranking provided by the CARS provides a strong basis for extracting meaningful scores from the medical record. Another important potential limitation is the reliance on nursing rather than physiological assessment of sleep duration, though this has been shown to be a reasonably accurate measure in psychiatric inpatients [27]. In addition, this paper focused on sleep duration as measured by the number of hours, and did not measure sleep quality either subjectively or in terms of sleep efficiency, i.e., the ratio of time spent asleep to the time spent in bed. Finally, there was no assessment of depressive symptoms and thus the prevalence and possible effects of mixed episodes, as well as the course of resolution of mixed depressive symptoms, were not accounted for.

## Conclusions

In sum, inpatient treatment of acute manic episodes is effective, with dissociable trajectories for improvement in sleep vs. improvement in mania and psychosis. Thus, both early normalization of sleep with continuing symptoms of mania and psychosis, and normalization of manic and psychotic symptoms with continuing sleep disturbance warrant continuation of treatment of the index manic episode.

## Ethics approval and consent to participate

This retrospective chart review study was approved by the institutional review board of the Beth Israel Medical Center of New York. Informed consent was waived due to the retrospective design of the study.

## Availability of data and materials

The data cannot be shared due to confidentiality issues.

## Abbreviations

BIMC: Beth Israel Medical Center of New York
BZD: Benzodiazepine
CARS-M: Clinician-Administered Rating Scale for Mania
FGAs: First generation antipsychotics
LOS: Length of stay
MS: Mood stabilizer
SGAs: Second generation antipsychotic
SUD: Substance use disorder
